# The hidden costs of ‘free’ treatment: A cross-sectional study of patient-incurred costs for daily methadone maintenance treatment in Nairobi, Kenya

**DOI:** 10.1371/journal.pmen.0000383

**Published:** 2025-09-02

**Authors:** Tina Wakukha Masai, Mary Wangari Kuria, Olatunde Olayinka Ayinde, Martine Odhiambo Oleche

**Affiliations:** 1 Department of Psychiatry, School of Medicine, Faculty of Health Sciences, University of Nairobi, Nairobi, Kenya; 2 Department of Psychiatry, College of Medicine, University of Ibadan, Ibadan, Nigeria; 3 Department of Economics and Development Studies, College of Humanities and Social Sciences, University of Nairobi, Nairobi, Kenya; University of Sunderland, UNITED KINGDOM OF GREAT BRITAIN AND NORTHERN IRELAND

## Abstract

Methadone Maintenance Treatment is one of Kenya’s evidence-based interventions and is stipulated in national policies and guidelines as both a harm reduction and addiction management intervention for opioid use disorder. It is provided free of charge at public health facilities where patients are required to come daily for treatment. Despite being free, patient-incurred costs such as transport, lost income and other daily expenses may pose barriers for treatment continuity, particularly in low resource settings such as Kenya. This cross-sectional study examined patient-incurred costs of daily methadone maintenance treatment (MMT) at a national teaching and referral hospital in Nairobi, Kenya. A total of 249 patients (80.32% male, 19.68% female) were surveyed using a socio-demographic questionnaire and a modified Drug Abuse Treatment Cost Analysis Program: Client (Outpatient) Version. Descriptive statistics and non-parametric tests were used to explore the association between patient demographic characteristics and treatment costs. Key findings showed a median age 40 years (IQR: 33–45), with 83.53% having a monthly household income $134.42. Patients travelled an average distance of 10 km daily and spent approximately 24 hours weekly seeking treatment. The median total direct cost was $ 22.6 per month (IQR: $13.2 – 37.6), while the median monthly income loss was $26.9 (IQR: $13.4 - $43.7). Female gender, higher education levels, household incomes, and current employment were significantly associated with greater costs and income losses (p < 0.05). Although MMT is free in Kenya, patients spent considerable time and opportunity costs in seeking treatment potentially compromising treatment adherence and long-term treatment outcomes. These findings suggest a need for decentralized services, take home dosing, and integrated livelihood programs to enhance MMT accessibility and effectiveness in resource-limited settings.

## Introduction

Methadone Maintenance Treatment (MMT) is an evidence-based intervention used for the management of Opioid Use Disorder. MMT has its origins in the United States dating back to the early 1960s where methadone was used as the primary pharmacotherapy. Methadone is a full opioid agonist which, when taken in optimal doses, reduces cravings and withdrawals associated with opioid use. Its 36–48-hour half-life and sustained plasma levels make it suitable for maintenance treatment [1]. This combined with various clinical and psychosocial interventions forms the package of care in the delivery of MMT.

Studies have demonstrated the efficacy of MMT in reducing opioid use and overdose risk, improving treatment retention, and decreasing HIV and hepatitis C transmission among people who inject drugs [2–4]. It has also been linked to reductions in criminal activity, improved social functioning, increased employment [5], and reduced costs associated with managing drug use-related conditions [4]. In Kenya, Methadone is classified as a First Schedule narcotic as per the Narcotics, Drugs and Psychotropic Substances (Control) Act of 1994 amended 2022, necessitating stringent regulations surrounding its distribution and use. This has resulted in treatment models that require patients to come into treatment daily, posing barriers to access and retention [6,7].

MMT in Kenya was introduced in 2014 as part of a comprehensive harm reduction package through the Ministry of Health and implemented through donor funding by the United States President’s Emergency Plan for AIDS Relief through the Centre for Disease Control (CDC) and United States Agency for International Development (USAID) [8,9]. Despite its benefits, access to MMT in Kenya is hindered by various barriers, including financial constraints. While provided free of charge in Kenya, patients incur direct and indirect costs related to transportation, opportunity costs due to time spent seeking treatment, and other associated expenses [10,11]. These patient costs can pose a significant treatment burden impacting treatment adherence and outcomes [12–15]. To assign a quantitative figure on patient costs, the study aimed to estimate the patient-incurred costs of seeking daily treatment for patients on methadone maintenance treatment in Nairobi, Kenya.

## Method

### Ethics statement

Ethical approval was obtained from the Kenyatta National Hospital-University of Nairobi Ethics Research Committee (KNH-UoN ERC - KNH/ERC/R/155) and the Mathari National Teaching and Referral Hospital Continuous Medical Education Department (MNTRH-CMED). A research permit was also obtained from the National Commission for Science, Technology & Innovation (NACOSTI). In addition to obtaining informed consent from all participants, research assistants signed confidentiality agreements regarding information received during the data collection process.

### Study design and population

This cross-sectional study used a patient perspective to examine patient-incurred costs associated with daily treatment attendance. The study was conducted at the Mathari Medically Assisted Treatment (MAT) clinic within Mathari National Teaching and Referral Hospital, a level 6 psychiatric referral facility in Kenya. Recruitment period spanned between 28^th^ December 2020 and 16^th^ January 2021 and was conducted based on the following inclusion criteria: active patients attending the clinic daily for treatment, had been on treatment for a minimum of 1 year at the time of the study, and were aged between 18–65 years, with a male-female ratio proportional to the demographic split of patients seeking services at the facility.

Out of 985 enrolled patients (860 males, 125 females), 605 (534 males, 71 females) were active at the time of the study. Using systematic random sampling from a de-identified line list of enrolled clients since 2014, the study applied the Yamane Formula (1967) with a 5% margin of error to arrive at a final sample size of 249 (200 males, 49 females).

### Measurements/Variables

A socio-demographic questionnaire (SDQ) was used to collect socio-demographic information from the participants, while patient cost data was collected using a modified version of the Drug Abuse Treatment Cost Analysis Program (DATCAP): Client (Outpatient) Version [16], which is designed for out-patient methadone maintenance treatment.

The SDQ captured independent variables such as age, sex, marital status, education level, number of children, employment status, and household income. Income disaggregation was informed by national surveys conducted by the Kenya National Bureau of Statistics (KNBS), with the $134.42 threshold for lower income groups representative of the 75th percentile of Kenyan household income per KNBS 2024 data [17]. It also aligns with national poverty benchmarks used in health economic studies in Kenya.

The modified DATCAP measured the dependent variables, focusing on direct and indirect patient costs of seeking treatment. Direct patient costs in this study comprised of daily round-trip transport expenses and other treatment-related costs such as child care, meals, or payments to others for covering work responsibilities [18,19]. Indirect patient costs comprised of quantified productivity losses as a result of lost or forgone income due to illness or treatment-seeking. In this study indirect patient costs comprised of time costs and income loss from treatment attendance [18,19] and were captured through self-reported measures of time spent, including daily travel time to and from the clinic, frequency of clinic visits per week, and time spent at the clinic (encompassing queuing, receiving services, relaxing within the facility and socializing). Total time spent seeking treatment was computed by summing travel time and time at the clinic. The tool also captured direct financial assistance received to seek treatment. Direct financial assistance comprised of monetary support provided directly to patients by others (family, friends or organizations) specifically to offset direct patient costs including transportation expenses or other out-of-pocket treatment-related expenses.

Distance travelled to the clinic was self-reported in kilometres. Data from the modified DATCAP was also used to approximate lost income from seeking treatment.

### Data collection procedures

Data collection was conducted by trained research assistants who administered the questionnaire using Kobo Toolbox. To identify eligible participants, a line list of enrolled clients was obtained and filtered to include only those active in care, defined as patients attending the clinic daily for treatment and having been on treatment for at least one year at the time of the study. The tool was pre-tested with a sample of 30 patients to ensure sensitivity, and the final tool was then coded in Kobo Toolbox for data collection. Informed consent was obtained from all participants prior to data collection. Data security was ensured through encrypted data transmission and secure, password-protected storage.

### Data analysis

Data was exported from Kobo Toolbox and resulting spreadsheets were cleaned in Microsoft Excel. The data was then uploaded to Stata BE 18 (Stata Corporation, College Station, TX) for further cleaning and analysis. Descriptive statistics were used to summarize the characteristics of the study population. For continuous variables such as age, distance travelled, and time spent seeking treatment, the study calculated measures of central tendency (mean and median) and dispersion (standard deviation and range). Categorical variables, such as gender, education level, and marital status, were summarized using frequencies and percentages. Direct patient costs were computed using the formula:


Direct\ Patient\ Costs=Transport\ Costs+Other\ Treatment\ Related\ Expenses

For all analyses, a p-value of less than 0.05 was considered statistically significant. Data was checked for independence of observations and equal variances and given the observed right-skewed distributions of key variables (Total Direct Cost and Income Loss), the study employed non-parametric tests. Mann-Whitney U test was used for comparisons between two groups (e.g., gender, marital status, household income categories) while the Kruskal-Wallis test was used for comparisons among three or more groups (education levels, age categories, employment status). These non-parametric tests were applied consistently for both Total Direct Cost and Income Loss analyses to examine associations with socio-demographic factors. Additional costs incurred to seek treatment were analysed using the same non-parametric approach. No outliers were removed from the datasets as they were deemed valid observations representative of the population of study.

Time spent seeking treatment was calculated by summing travel time and time spent at the clinic. Income lost due to seeking treatment was based on daily income against days worked. It then factored the equivalent days lost from seeking treatment to arrive at aggregate income loss.

## Results

### Characteristics of study participants

A total of 249 participants were interviewed, comprising 200 (80.3%) males and 49 (19.7%) females. The median age was 40 years (IQR: 33–45 years), with a range from 22 to 64 years. The majority of participants (43%, n = 107) were married or cohabiting, while 57. % (n = 142) reported other relational statuses (single, divorced, separated, or widowed).

Family size varied among participants, with a median of 2 children (IQR: 1–2). Notably, 16.5% (n = 41) reported having no children, while 8.4% (n = 21) had 4 or more children.

Education levels were diverse, with nearly half (49.4%, n = 123) having attained primary education, while 34.9% (n = 87) had secondary education, and 12.5% (n = 31) had tertiary qualifications. Eight participants (n = 3.2%) reported no education.

Employment status also varied considerably among participants. The largest group (31.7%, n = 79) reported never being employed, followed by those currently employed (25.7%, n = 64). A significant portion (24.5%, n = 61) had been unemployed for longer than 12 months, while 16.5% (n = 41) had been employed within the past year. A small fraction (1.6%, n = 4) became unemployed just before entering treatment.

At the time of analysis, the conversion rate was $1 = Kes 148.8. The economic status of participants was predominantly low, with a median monthly household income falling in the ≤ $134.4 category. This lowest income bracket encompassed 83.5% (n = 208) of participants, underscoring the economic challenges faced by the majority of patients seeking MMT. Only 16.5% (n = 41) reported incomes above $134.4, with a small minority (1.6%, n = 4) earning $672.13 or more per month. This distribution mirrors national patterns where approximately 75% of Kenyan households fall below this income threshold.

Table 1 presents a detailed breakdown of the demographic characteristics of the study participants, including frequencies, percentages, and where appropriate, median values with interquartile ranges. The complete de-identified dataset is available in S1 Data.

**Table 1:** Demographic characteristics of patients on MMT at mathari clinic.

Demographic Characteristics: n = 249	Frequency	Percent
**Sex**		
Male	200	80.32%
Female	49	19.68%
**Age Group**		
Less than 35 years	74	29.72%
35 to 44 years	108	43.37%
45 years and above	67	26.91%
**Marital status**		
Married or cohabiting	107	42.97%
Other Status	142	57.03%
**Number of Children**		
None	41	16.47%
1	72	28.92%
2	76	30.52%
3	39	15.66%
≥4	21	8.43%
**Formal Education**		
No Education	8	3.21%
Primary	123	49.41%
Secondary	87	34.9%
Tertiary	31	12.45%
**Employment Status**		
Never employed	79	31.73%
Longer than 12 months ago	61	24.50%
During the past 12 months	41	16.47%
Just before entering treatment but not currently	4	1.61%
Currently Employed	64	25.70%
**Household Income**		
≤ $134.42	208	83.53%
≥ $134.43	41	16.47%

### Patient-incurred costs of seeking Methadone maintenance treatment

To understand patient-incurred costs of seeking daily treatment, the study focused on total direct costs incurred and income lost from seeking treatment.

### Direct patient cost of seeking treatment

To assess the financial burden on patients, the study examined the direct patient costs associated with seeking treatment. These costs comprised of transport expenses and other treatment-related expenses. Given that MMT is provided free of charge in Kenya’s public health system, direct patient costs primarily consisted of transportation and other treatment-related expenses, with no significant medical costs reported by participants. Additionally, direct financial assistance received by patients was measured separately.

Direct patient costs were computed using the formula:


Direct\ Patient\ Costs=Transport\ Costs+Other\ Treatment\ Related\ Expenses

Analysis of the direct patient costs revealed a right-skewed distribution, indicating that while most participants incurred moderate costs, a subset faced significantly higher expenses. Among those who incurred direct costs (n = 217, 87%), the median total direct cost was $22.6 (IQR: $13.2 - $37.6), with costs ranging from $2.2 to $141.1 per month.

Data from various countries in sub-Saharan Africa indicate that approximately 10% of income goes to transport costs [20–22]. This study used 10% as the benchmark of the transport costs relative individual income. Among participants who incurred costs, 162 (65%) exceeded this threshold. Given that 83.5% of patients also reported a monthly household income below $134.4, it is likely that a significant proportion of individual and household income is going to treatment-related transportation costs.

In addition to transportation costs, 161 (65%) participants reported incurring other treatment related expenses related to accessing the MMT program. These additional expenses included childcare while seeking treatment, buying meals or sweets due to cited effects of medication (bitter taste, hunger), and compensating others for covering work responsibilities during treatment visits. The median additional costs incurred, amounted to $5.7 per month (IQR: $3.8 - $14.1), with some patients reporting additional costs as high as $47 per month indicating wide disparities among patients. While females (n = 34) showed higher median additional costs compared to males (n = 127) at $2.8 vs $1.6, this difference was not statistically significant (p = 0.4).

Additionally, 32 patients (12.9%) received direct financial assistance from others to help with treatment attendance. To further understand the distribution of these costs, we analysed their association with various demographic characteristics. Table 2 presents the associations between direct patient costs and demographic characteristics.

**Table 2:** Association between direct patient costs and demographic characteristics among participants incurring costs (n = 217).

Demographic characteristic	Median cost (IQR) USD	Test statistic	p-value
**Gender**		U = 3535.5	0.5
Male	22.6 (13.2 – 37.6)		
Female	24.5 (16.1 – 37.6)		
**Age**		H = 1.2	0.5
≤ 34 years	24.5 (13.2 – 37.6)		
35 - 44 years	22.6 (13.2 – 37.6)		
≥ 45 years	22.6 (13.2 – 37.6)		
**Marital Status**		U = 5412.5	0.3
Married or cohabiting	24.5 (13.2 – 37.6)		
Other status (single, divorced, widowed, separated)	22.6 (13.2 – 37.6)		
**Number of Children**		H = 1.8	0.4
No children/one child	22.6 (13.2 – 37.6)		
2 children	22.6 (13.2 – 37.6)		
≥ 3 children	24.5 (15.4 – 37.6)		
**Formal Education**		H = 16.4	**0.02**
Primary	22.6 (9.4 – 32.0)		
Secondary	24.5 (18.8 – 37.6)		
Tertiary	37.6 (20.4 – 56.6)		
**Employment Status**		U = 5892.0	0.4
Never employed - employed longer than 12 months ago	22.6 (13.2 – 37.6)		
Employed in past 12 months – currently employed	24.5 (13.2 – 37.6)		
**Household Income**		U = 2622.5	**0.01**
Less than $134.4	22.6 (13.2 – 37.6)		
≥$134.4	28.2 (18.8 – 47.0)		

The results indicate significant associations between total direct costs and both education level (p = 0.02) and household income (p = 0.01). Participants with tertiary education and those with higher household incomes tended to incur higher direct costs. Among the 217 participants who incurred costs, 35 (16%) had zero transportation expenses but still incurred other treatment-related costs such as childcare, meals, or work coverage payments.

### Indirect patient costs: Time and income lost due to seeking treatment

To look at the income lost from seeking treatment per month, the study computed the travel time, frequency of clinic visits and time spent seeking treatment.

Findings revealed a significant time commitment for patients. On average, participants travelled 9.94 kilometres (km) daily to reach the clinic, with distances ranging from 1 km to 68 km. Most patients commuted about 2 km. to get to the clinic. This journey took an average of 1.48 hours per round trip, with many patients spending up to 2 hours travelling each day. Patients visited the clinic 7 days a week, amounting to approximately 10.36 hours of travel time (to and from the clinic) weekly. Once at the clinic, they spent approximately 13.6 hours per week engaged in various activities, including queuing, receiving treatment, relaxing, socializing, and a proportion of those who incurred transport costs sometimes waiting for public transport fares to decrease. In total, patients spent nearly 24 hours per week seeking treatment, equivalent to 96 hours or *four full days* each month.

Of note is the fact that while travel time was similar among both low income and mid-high-income groups (44 minutes vs 45 minutes), travelled distance was on average 2.3km more for the mid-high-income groups suggesting slightly longer distances travelled to receive treatment.

Although not all time spent seeking treatment translates to lost income, the study sought to estimate what lost income would compute to if earnings were foregone in order to seek treatment. Given that majority of patients (170; 68.3%) lost income to seek for treatment, and 2/3 of the participants earn daily wages, the impact is substantial. The study based its analysis on those who reported employment history, excluding those who have never been employed. The study estimates that losing the equivalent of 4 working days in a month resulted in income lost ranging from $2.7 to $ 336 per patient. Among participants with employment history (170, 68.3%), the median income loss per month was $26.9 (IQR: $13.4 - $43.7) overall, with significant variation by gender: $21.8 (IQR: $13.4 - $43.7) for males and $40.3 (IQR: $21.8 - $53.8) for females, representing a statistically significant difference (p < 0.001) showing females incurred greater income losses.

Table 3 provides a detailed breakdown of the associations between income lost and demographic characteristics.

**Table 3:** Association between income lost and demographic characteristics (n = 170).

Demographic characteristic	Median Income Loss (IQR) in USD	Test statistic	p-value
**Gender**		U = 1476.0	**<0.001**
Male	21.8 (13.4 – 43.7)		
Female	40.3 (21.8 – 53.8)		
**Age**		H = 2.8	0.2
≤ 34 years	25.2 (10.9 – 43.7)		
35 - 44 years	26.9 (16.1 – 53.8)		
≥ 45 years	21.7 (13.4 – 40.3)		
**Marital Status**		U = 2845.0	0.6
Married or cohabiting	26.9 (13.4 – 48.3)		
Other status (single, divorced, widowed, separated)	24.2 (13.4 – 43.7)		
**Number of Children**		H = 1.2	0.5
No children/one child	26.9 (13.4 – 53.8)		
2 children	23.5 (13.4 – 40.3)		
≥ 3 children	26.9 (16.1 – 43.7)		
**Formal Education**		H = 8.5	**0.01**
Primary	18.8 (8.1 – 43.7)		
Secondary	26.9 (15.5 – 43.7)		
Tertiary	31.8 (25.2 – 60.5)		
**Employment Status**		U = 2156.0	**<0.001**
Current Employment – Employed in the past 12 months	31.9 (16.1 – 53.8)		
Past Employed (> 12 months ago)	18.8 (13.4 – 28.6)		
**Household Income**		U = 1845.0	**<0.001**
Less than $134	21.8 (13.4 – 43.7)		
≥$134.4	53.8 (26.9– 100.8)		

Significant associations were observed between income loss and gender (p = < 0.001), education level (p = 0.0), household income (p < 0.001), and employment status (p < 0.001). Female participants and participants with higher education levels, higher household incomes, and current or recent employment tended to report greater income losses.

While this study did not directly measure treatment retention, the substantial costs incurred by patients suggest potential implications for long-term engagement in MMT. The median direct patient cost of $22.6 (IQR: $13.2 - $37.6) represents a significant proportion of income for many patients, particularly given that 83.5% of participants reported monthly household incomes below $128. Furthermore, the median monthly income loss of $26.9 (IQR: $13.4 - $43.7) adds to the economic burden. These financial pressures could potentially impact patients’ ability to consistently engage with daily treatment over extended periods.

## Discussion

This study assessed the patient costs of daily MMT at a public health facility in Nairobi City County, Kenya. The findings revealed a predominantly middle-aged population with lower education status and low combined household incomes, consistent with other studies [23–25].

The low male-to-female ratio in the sample reflects both the active numbers at the clinic and the nationally reported ratio of male-to-female injecting drug users, with similar patterns observed across multiple regions in and out of Africa [26–29]. This ratio further illustrates the difficulty in reaching the female population due to misinformation, multi-faceted stigma, negative perceptions associated with Methadone use, and barriers to access treatment [26,29,30]. While there were no statistically significant differences by gender for direct patient costs, income losses were substantial compared to men. Other studies globally and in East Africa have highlighted significant barriers faced by women in relation to treatment-related costs such as transportation, child care, domestic responsibilities and expectations on caregiving, all of which increase economic inequities for women, potentially impacting treatment access and cost [14,31–36]. The highlighted challenges suggest the need for not only flexible enrolment models, but also women-friendly retention models that address the specific needs of women with opioid use disorder.

Analysis revealed significant associations between direct patient costs and both education level and household income. Participants with tertiary education and those with higher household incomes tended to incur higher direct costs. While other studies did not directly link costs to education and household income, findings by Wanyoike et al. [13] indicated the dual impact of employment and income on MMT retention. Lack of employment or income meant lack of transport for daily clinic visits while employment despite income meant a schedule that interfered with treatment visits. Studies in Asia highlighted the impact of higher education, lower income and employability on retention with inferences to patient costs with MMT interfering with social and occupational responsibilities [15,37,38]. In Malaysia, transport costs were seen to be higher and linked to employability [39]. Applied to the Kenyan population given the slightly higher distances travelled by participants with higher income, it could be inferred that participants with higher education and employment status are able to reside further away from the clinics and gain meaningful employment resulting in higher costs associated with accessing treatment daily.

Notably, 65.1% of participants exceeded the 10% recommended threshold of income spent on transportation costs [20–22]. Although studies identified did not quantify transport cost relative to individual and household income, regional studies from East Africa qualitatively highlight transport barriers and their impact on treatment retention [34–35]. Bowser et al. [40] linked transport as one of the significant factors in direct patient costs on MMT particularly due to daily visits. Knight et al. [34] found that travel distance and transport costs were significant factors affecting treatment retention in Tanzania, while Kiburi et al. [14] highlighted the link between lack of transport and missed dosing. Johns et al. [41] demonstrated that journeys exceeding 30 minutes to MMT clinics significantly increased attrition risk in Vietnam and a study from Malaysia indicated that out-of-pocket costs for MMT came to 35% of patient income [39].

The study further demonstrated that income loss due to seeking treatment daily was significantly associated with gender, education level, household income, and employment status with those with females, those with higher education levels and household incomes, and current employment reporting greater income losses. This may be influenced by ability to access jobs that position them at a higher income level. Daily treatment visits could thus result in greater income loss particularly for clients earning daily wages. These findings can be inferred from willingness to pay studies where higher education and higher income has been associated with increased willingness to pay [42] and retention studies where high income, more educated and employed persons are less likely to be retained on treatment [37].

Findings from Norway by Pedersen et al. [43] computed patient incurred costs and found that patients who received oral methadone in Opioid Agonist Treatment (OAT) clinics spent 17.8 hours per month, amounting to an implied value of €426.3 (approximately $419.3). This contrasted with findings from this study that saw patients spending five times more time per month seeking treatment (96 hours) and with a widely disparate income loss ranging from $0.8 to $336 per patient. This wide range in income loss highlights the disproportionate impact of treatment seeking on different socioeconomic groups even when compared to high-income countries. It further has implications with treatment rehabilitation and employability due to lost productivity and challenges balancing MMT with occupational and social responsibilities [13,14,43,44].

The client DATCAP, while a dated tool, remains relevant for the quantification of patient-incurred costs of out-patient MMT. While recent studies using the client DATCAP for out-patient MMT settings are lacking, other studies that have quantified patient-incurred costs use varied tools, while others only compute specific patient-costs such as medical out-of-pocket costs which only paint a partial picture of patient-incurred costs [43,45].

The substantial patient costs associated with daily MMT, as revealed in this study, have important implications for treatment retention. While retention was not directly measured, the financial burden identified—both in terms of direct costs and income loss—could potentially act as a barrier to long-term treatment engagement. Patients facing high relative costs, particularly those from lower-income households who are the majority, may struggle to maintain consistent daily attendance [37,46]. This economic strain could lead to missed doses or treatment discontinuation, undermining the effectiveness of MMT programs. Moreover, the time commitment required for daily clinic visits may conflict with employment opportunities, further complicating the balance between treatment adherence and financial stability [47].

The study is however not without limitations. The cross-sectional design, while effective in providing a snapshot of patient-incurred costs, is limited in accounting for variation in costs over time and its net effect on treatment in the long-term. Future longitudinal studies can address these limitations. With the study conducted in an urban centre, further studies should incorporate a rural-urban mix that accurately reflects the different geographical contexts in Kenya. Despite the fact that the study was conducted in one site, sampling was stratified to ensure representation from varied regions in the county. While the sample size of women was representative of the clinic population, it was relatively low; thus, future studies should not only assess quantitative measures but also allocate and measure qualitative variables among women to improve insights on provision of equitable services for women on MMT. Finally, given the study relied on self-report for costs, distance travelled and time spent seeking treatment, this could have resulted in social desirability bias. Future studies could implement mixed approaches minimize bias when arriving at cost data.

## Recommendations

These findings underscore the need for retention-focused strategies that address the economic challenges faced by patients. Such strategies could include decentralized service delivery to reduce travel costs, flexible dosing schedules to minimize work conflicts, scale-up of integrated or mobile sites and integrated livelihood programs to offset the financial burden of treatment. These strategies could help mitigate these patient costs and improve treatment accessibility and outcomes by addressing the emergent needs of persons in SUD treatment [48].

The results of this study further highlight the need for policymakers and healthcare providers to consider the full spectrum of costs borne by patients when designing and implementing MMT programs. Furthermore, the finding that those with higher socioeconomic status face greater absolute financial burdens suggests a need for tailored support mechanisms. While higher-income patients might be better able to absorb these costs in the short term, the long-term sustainability of their treatment engagement could be at risk. Conversely, while lower-income patients show lower absolute costs, the relative impact on their limited resources may be more severe, potentially leading to treatment discontinuation.

Our findings, when linked to the global context of MMT programs, reveal both shared challenges and unique aspects of the Kenyan experience. The patient costs related to transportation and lost productivity echo findings from different parts of the world [11,15,37,39]. They further lend additional insights to previous studies conducted in Kenya that highlight transport barriers but do not assign a tangible cost [13,14,35].

The time burden associated with daily clinic visits, a significant finding in our study, aligns with global calls for more flexible MMT delivery models such as take-home dosing and community-based dispensing models.

## Conclusion

Findings of this study demonstrated significant direct and indirect patient costs associated with receiving daily MMT. Of note is the fact that productivity losses account to approximately 4 days per month with significant gender disparities in income loss. This burden was borne most significantly by those with higher levels of education, income and employment.

Future research should expand to rural contexts where MMT is provided, conduct longitudinal assessments of patient costs on long-term treatment adherence and outcomes, evaluate societal costs to paint a wider picture of true total costs and evaluate the cost-effectiveness of different MMT delivery models in the Kenyan context. Addressing these indirect costs is essential for enhancing the accessibility, effectiveness, and long-term success of MMT programs in resource-limited settings like Nairobi, Kenya.

## Supporting information

S1 Data Raw de-identified dataset of patient-incurred costs for methadone maintenance treatment study conducted in Nairobi, Kenya. (XLSX)
